# The Genetic Structure of *Leishmania infantum* Populations in Brazil and Its Possible Association with the Transmission Cycle of Visceral Leishmaniasis

**DOI:** 10.1371/journal.pone.0036242

**Published:** 2012-05-11

**Authors:** Gabriel Eduardo Melim Ferreira, Barbara Neves dos Santos, Maria Elizabeth Cavalheiros Dorval, Tereza Pompilio Bastos Ramos, Renato Porrozzi, Alexandre Afranio Peixoto, Elisa Cupolillo

**Affiliations:** 1 Laboratório de Pesquisa em Leishmaniose, Instituto Oswaldo Cruz – Fundação Oswaldo Cruz, Rio de Janeiro, Brasil; 2 Laboratório de Biologia Molecular de Insetos, Instituto Oswaldo Cruz – Fundação Oswaldo Cruz, Rio de Janeiro, Brasil; 3 Centro de Ciências Biológicas e da Saúde, Universidade Federal do Mato Grosso do Sul – Mato Grosso do Sul, Brasil; 4 Secretaria de Estado de Saúde do Mato Grosso, Superintendência de Vigilância em Saúde, Coordenadoria de Vigilância em Saúde Ambiental, Mato Grosso, Brasil; University of Nottingham, United Kingdom

## Abstract

*Leishmania infantum* is the etiologic agent of visceral leishmaniasis (VL) in the Americas, Mediterranean basin and West and Central Asia. Although the geographic structure of *L. infantum* populations from the Old World have been described, few studies have addressed the population structure of this parasite in the Neotropical region. We employed 14 microsatellites to analyze the population structure of the *L. infantum* strains isolated from humans and dogs from most of the Brazilian states endemic for VL and from Paraguay. The results indicate a low genetic diversity, high inbreeding estimates and a depletion of heterozygotes, which together indicate a predominantly clonal breeding system, but signs of sexual events are also present. Three populations were identified from the clustering analysis, and they were well supported by *F* statistics inferences and partially corroborated by distance-based. POP1 (111 strains) was observed in all but one endemic area. POP2 (31 strains) is also well-dispersed, but it was the predominant population in Mato Grosso (MT). POP3 (31 strains) was less dispersed, and it was observed primarily in Mato Grosso do Sul (MS). Strains originated from an outbreak of canine VL in Southern Brazil were grouped in POP1 with those from Paraguay, which corroborates the hypothesis of dispersal from Northeastern Argentina and Paraguay. The distribution of VL in MS seems to follow the west-east construction of the Bolivia-Brazil pipeline from Corumbá municipality. This may have resulted in a strong association of POP3 and *Lutzomyia cruzi*, which is the main VL vector in Corumbá, and a dispersion of this population in this region that was shaped by human interference. This vector also occurs in MT and may influence the structure of POP2. This paper presents significant advances in the understanding of the population structure of *L. infantum* in Brazil and its association with eco-epidemiological aspects of VL.

## Introduction

*Leishmania infantum* Nicole 1908 is a causative agent of visceral leishmaniasis (VL) in endemic countries in Southern Europe, North Africa, West and Central Asia and the Americas [1]. This species belongs to the *L. donovani* complex [2], [3]. Recently, a study that compared microsatellite profiles [4] strongly supported the hypothesis that *L. infantum* was introduced in the New World during or after the Colonial period, and this introduction may have occurred more than once, as previously proposed [5], [6], [7], [8], [9].

American visceral leishmaniasis (AVL) has been documented from Northern Argentina to Southern United States [1], [7], [10], [11], [12]. Almost 70,000 cases from 21 Brazilian states were recorded between 1980 and 2008, and these cases primarily occurred in the Northeast Region [1], [11], [13].

Initially, AVL was associated with rural and poor peri-urban areas, but this disease is displaying a new pattern, and urban cases are becoming common [1], [14], [15], [16], [17], [18], [19]. Dogs are the main reservoirs of AVL, and shelters of domestic animals likely act as breeding sites or provide a niche for the maintenance of the vector population close to residences [14], [20], [21], [22], [23].

In Brazil, the dispersion of AVL has been associated with the intense human migrations that occurred from the Northeast, where 82.5% of the Brazilian cases occur [16], to the urban centers of the Central West and Southeast Regions (São Paulo [SP] and Minas Gerais States). The Leishmaniases are known to follow anthropic events, such as demographic expansion and migration, hydroelectric plants, road construction or gas pipelines [1], [17], [18], [24], [25], [26], [27].

Recently, the South Region experienced its first outbreak of canine visceral leishmaniasis, which may have been imported from neighboring endemic countries [28].

**Figure 1:**
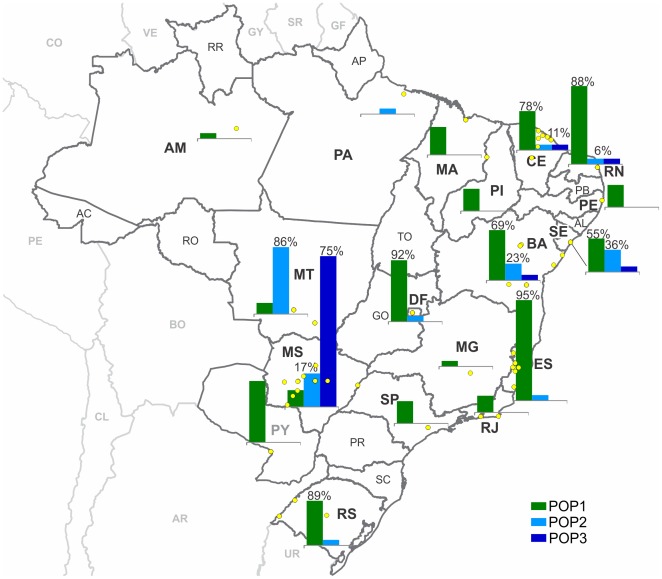
The geographic origin of *Leishmania infantum* strains and populations of STRUCTURE analysis. The yellow dots represent the locations of the collections of each analyzed strain. The graphics indicate the proportion numbers of the strains (Y axis) in each population (X axis). The assignment of the strains to a population was performed in the STRUCTURE analysis that was based on the profiles of 14 microsatellite markers. POP1 is a widespread population, and it is predominant in most of the foci. POP2 and POP3 are predominant in Central West Brazil where *Lutzomyia longipalpis* and *Lutzomyia cruzi* are involved in the transmission cycle of Visceral Leishmaniasis. The abbreviations for the Brazilian states are as follows (in bold): AM, Amazonas; BA, Bahia; CE, Ceará; DF, Distrito Federal; ES, Espírito Santo; MA, Maranhão; MG, Minas Gerais; MT, Mato Grosso; MS, Mato Grosso do Sul; PA, Pará; PE, Pernambuco; PI, Piauí; RJ, Rio de Janeiro; RN, Rio Grande do Norte; RS, Rio Grande do Sul; SE, Sergipe; SP, São Paulo. International country codes: AR, Argentina; BO, Bolivia; CL, Chile; CO, Colômbia; GF, French Guiana; GY, Guyana; PY, Paraguay; PE, Peru; SR, Suriname; UR, Uruguay; VE, Venezuela. See Table S1 for more details.

In most endemic areas of American countries, *Lu. longipalpis* is the main vector of *L. infantum* [29]. However, the relevance of secondary vectors has been discussed. In Colombia, the sand fly *Lu. evansi* was reported to be an alternate vector of *L. infantum* [30]. Recently, *Lu. migonei* was indicated as a putative vector in Pernambuco (PE), Brazil, based on the observation of its natural infection with *L. infantum* and the absence of *Lu. longipalpis* in the area [31]. In some Brazilian states from the West Region, such as Mato Grosso (MT) and Mato Grosso do Sul (MS), *Lu. cruzi* has been reported infected by *L. infantum* [32], [33], [34]. This sand fly species belongs to the *Lu. longipalpis* complex, and their overlapping distributions in many areas of the Central West [35], [36], [37], [38] might have important implications for the evolutionary process of vector speciation [39], [40], [41].

**Table 1:** Descriptive statistics of the *Leishmania infantum* MLMT profiles from of overall sample.

Locus	N	Repeat array	Size array	A	*H*_o_	*H*_e_	*F*_IS_
Lm2TG (L)	173	23/30	138/152	8	0.0578	0.4955	0.8836
TubCA (T)	172	9/10	80/82	2	0	0.0344	1
Lm4TA (M)	167	11/14	77/83	4	0.0719	0.4696	0.8474
Li41-56 (B)	170	10/11	90/92	2	0	0.0117	1
Li46-67 (C)	172	9/10	80/82	2	0.0116	0.0116	−0.0029^ns^
Li22-35 (E)	165	12/16	92/100	4	0.0121	0.1479	0.9183
Li23-41 (F)	162	12/17	77/87	3	0	0.0485	1
Li45-24 (G)	172	3/16	81/107	4	0.0058	0.0950	0.9390
Li71-33 (P)	171	10/12	103/107	3	0.0175	0.0630	0.7219
Li71-5/2 (Q)	173	9/9	110/110	1	0	0	0
Li71-7 (R)	171	12/14	92/102	3	0.0117	0.0117	−0.0015^ns^
CS20 (S)	173	18/18	83/83	1	0	0	0
kLIST7031 (K)	167	11/11	111/111	1	0	0	0
kLIST7039 (I)	168	14/17	205/211	3	0	0.0468	1
Overall	170			2.93	0.0135	0.1026	0.8691

*n*, the number of samples per locus; A, the number of alleles per locus; *H*_o_, the observed heterozygosity; *H*_e_, the expected heterozygosity; *F*_IS_, the inbreeding coefficient;

ns , non-significant values under confidence interval of 95%. Descriptive statistics for populations from STRUCTURE analysis is shown in Table S2.

In the Old World, there are many vector species of *L. infantum* (WHO, 2010). This *Leishmania* species is known to infect many sand fly vectors, even when strains from the same zymodeme (MON-1) were considered [42]. The *L. infantum* zymodeme 1 seems to be predominant in the Neotropics; however, based on a set of microsatellite markers, some Central American strains have been shown to be more similar to the European non-MON-1 [4]. This approach, which is known as multi-locus microsatellite typing (MLMT), is based on the polymorphism of short tandem repeats that have been widely applied in different organisms as a result of advances in fragment-size detection [43], [44], [45], [46]. Sets of multilocus microsatellite markers have been developed for *Leishmania* species, and they have proven to be highly sensitive for assessing genetic variation on an intra-zymodeme level [47], [48], [49], [50], [51], [52]. These sets of markers have improved studies on the population genetics and molecular epidemiology of VL in Mediterranean areas and in the Middle East [52], [53], [54], [55], [56], [57], [58], [59], [60], [61].

**Table 2:** The distribution of MLMT genotypes shared among *Leishmania infantum* strains by hosts and geographic origins.

SplitsTree genotype	Dog	Human
‘TYPE1’	ES (6)	
‘TYPE2’	ES (2)	
‘TYPE3’	ES (1)	ASU (1)
‘TYPE4’		BA (1); MG (1)
‘TYPE5’		ES (2)
‘TYPE6’	SP (2)	
‘TYPE7’	ASU (1)	SE (1)
‘TYPE8’	RS (2)	
‘TYPE9’		DF (1); SE (1)
‘TYPE10’	ASU (6); BA (3); CE (4); DF (1); MS (1); PE (2); PI (2); RJ (1); RN (3); RS (3); SP (2)	BA (2); CE (1); DF (5); ES (3); MA (2); MS (2); PE (2); RJ (2); RN (2); SE (3)
‘TYPE11’	ES (1); RS (1)	
‘TYPE12’	PI (2)	MA (2)
‘TYPE13’	ASU (3)	
‘TYPE14’	MT (1); RN (4)	RN (1)
‘TYPE15’	ES (1)	MS (1)
‘TYPE16’	MS (1)	MS (1); SE (2)
‘TYPE17’	MS (1); MT (4)	MS (1)
‘TYPE18’		DF (1); RN (1); SE (1)
‘TYPE19’	MT (1)	CE (1)
‘TYPE20’		MS (2)
‘TYPE21’		MS (2)
‘TYPE22’	MS (2)	CE (1); MS (15)

POP1, ‘TYPE1’ to ‘TYPE14’; POP2, ‘TYPE15’ to ‘TYPE19’; POP3, ‘TYPE20’ to ‘TYPE22’. Brazilian States: AM, Amazonas; BA, Bahia; CE, Ceará; DF, Distrito Federal; ES, Espírito Santo; MA, Maranhão; MG, Minas Gerais; MT, Mato Grosso; MS, Mato Grosso do Sul; PA, Pará; PE, Pernambuco; PI, Piauí; RJ, Rio de Janeiro; RN, Rio Grande do Norte; RS, Rio Grande do Sul; SE, Sergipe; SP, São Paulo. Paraguay: ASU, Asunción.

The number of strains per state for each genotype is shown in brackets.

See Table S1 for more details.

Previous microsatellite-based studies from New World *L. infantum* indicated the existence of two main populations; one population contains strains from Paraguay, Brazil, Colombia and Honduras and is composed of zymodeme MON-1, and a second population contains strains from Venezuela, Panama, Costa Rica and Honduras and is related to non-MON-1 zymodemes [4]. Substructures within the New World MON-1 population were also detected but failed to reach statistical significance. Furthermore, when the Neotropical and Old World strains were analyzed together, the first population resembled those from Portugal and Spain, from which the New World MON-1 strains likely originated [4], [62].

**Figure 2:**
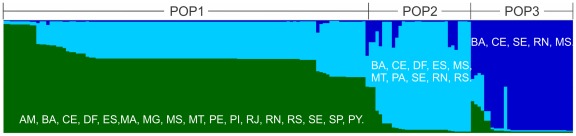
*Leishmania infantum* populations inferred from STRUCTURE analysis based on profiles of 14 microsatellites. The barplot was generated in Excel using the results of the aligned distribution of Q values from 10 for *K* = 3, which was generated using CLUMPP software. Evanno's method predicted that three was the most likely number of populations (Figure S1). The POP1 (n = 111) is composed of strains from all of the foci (with the exception of PA), and most of the strains contain traces from POP2. POP2 (n = 31) includes strains from 10 states, but it is predominantly observed in MT. POP3 (n = 31) is composed primarily of MS strains and of four strains from other states of Northeast Brazil. Abbreviations: AM, Amazonas; BA, Bahia; CE, Ceará; DF, Distrito Federal; ES, Espírito Santo; MA, Maranhão; MG, Minas Gerais; MT, Mato Grosso; MS, Mato Grosso do Sul; PA, Pará; PE, Pernambuco; PI, Piauí; RJ, Rio de Janeiro; RN, Rio Grande do Norte; RS, Rio Grande do Sul; SE, Sergipe; SP, São Paulo; PY, Paraguay.

In fact, MLMT has been demonstrated to successfully evaluate the population structure of *L. infantum*, which can help to elucidate epidemiological aspects, such as the spread of the parasites across endemic areas and the origin of VL outbreaks [4], [57], [62].

**Figure 3:**
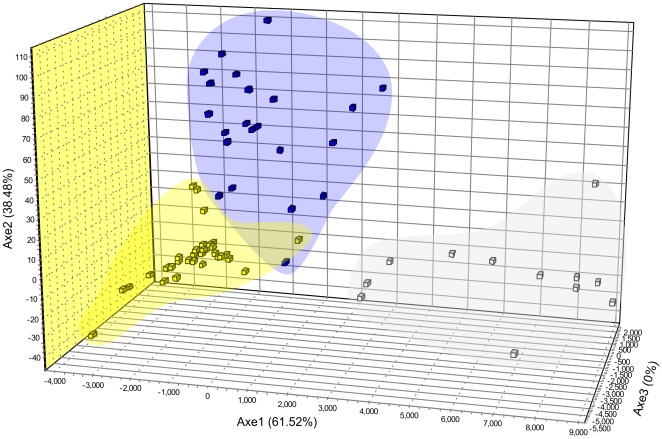
Factorial correspondence analysis (FCA) of the *Leishmania infantum* genotypes. Spacial distribution of strains after FCA of microsatellite genotypes. Blue dots, POP1; yellow dots, POP2; grey dots, POP3.

In this study, we described the *L. infantum* population structure from a representative sample of Brazilian endemic areas. We increased the sampling from a previous study [4] and included strains from outbreaks of exclusive CVL in South Brazil and from re-emerging foci in Central West Brazil. The sample locations also include areas in which vectors other than *Lu. longipalpis* may participate on the transmission cycle. In addition, some geographical hypotheses were corroborated by the observed MLMTypes. Human influence on the dispersion of genotypes and expectations for further VL studies were also discussed.

**Figure 4:**
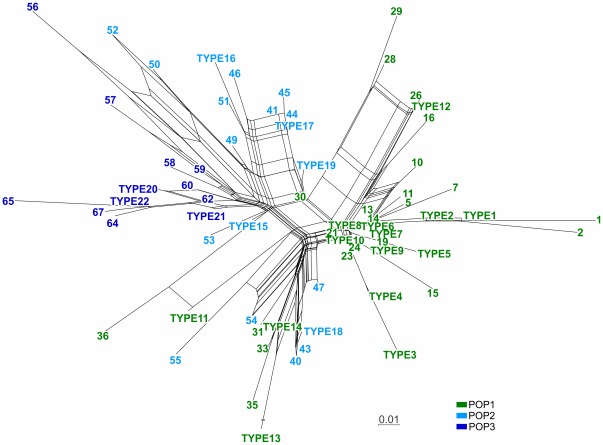
Neighbor-net constructed on SplitsTree software employing the chord distance values among the *Leishmania infantum* genotypes. Identical genotypes for the 14 microsatellite markers were grouped and are represented by “TYPEs” (see Table S1). The distribution of the splits shows the same populations that were determined by the STRUCTURE analysis (Figure 2); some genotypes from POP2 are closer to POP1, and others are closer to POP3.

## Materials and Methods

### Sample set

The samples were obtained from the *Leishmania* collection of the Oswaldo Cruz Institute (CLIOC – http://clioc.fiocruz.br). We included 162 strains from most Brazilian states and 11 from Asunción, Paraguay (Figure 1) that were typed by MLEE as *L. infantum* zymodeme 1. Twenty strains included here were previously analyzed by Kuhls et al. [4] (Table S1). Eighty-four of the samples were from humans, 87 were from dogs, one was from opossum (*Didelphis marsupialis*) and one was from fox (*Cerdocyon* sp.). Table S1 provides the sample information, including the international code [1], CLIOC code (IOC/L) and geographic origin.

### DNA extraction and PCR amplification

The DNA was isolated and extracted using the Wizard™ Genomic DNA Purification System (Promega, Madison, WI, USA) following the manufacturer's protocol; the DNA was stored at 4°C. The PCRs were performed with 14 primer pairs (Table 1) that were previously described for studies of species from the *L. donovani* complex [59]. The forward primers of each pair were conjugated with 6FAM or HEX fluorophores. Fragment-length screening was performed using Mega BACE 1000 (GE Healthcare) or ABI 3100XL (Applied Biosystems) from the Fragment Analysis Platform of the Oswaldo Cruz Institute (Plataforma Genômica – Sequenciamento de DNA – PDTIS/FIOCRUZ; RPT01A) using ET-ROX 400 (GE Healthcare) and GeneScan 500 ROX (Applied Biosystems), respectively, as the size standards.

The length analyses were performed on the Genetic Profile 2.2 (GE Healthcare) and Peak Scanner™ Software v1.0 (available at http://www.appliedbiosystems.com accessed on October 14, 2011). The results from both software packages were standardized based on strains of known sizes.

### Data analysis

The STRUCTURE 2.3 software [63] was used to cluster individuals into populations with a model-based approach. The assigned proportion of each individual belonging to each population (membership coefficient Q) was estimated using Bayesian statistics and Markov Chain Monte Carlo simulations with a 100,000 iterations burn-in period that was followed by further 1,000,000 steps. For each value of *K* (from 1 to 10), 10 iterations were performed to estimate values of Δ*K* [64], which were implemented on Structure Harvester v0.6.1 (Earl, 2011; available at http://taylor0.biology.ucla.edu/structureHarvester/accessed on October 14, 2011). The software CLUMPP 1.1.2 [65] was used to deal with the “label switching” performing alignments of the Q values for the chosen number of populations. The barplots of the CLUMPP outfiles were visualized using the Excel software (Microsoft). This clustering approach was used as a base for the population genetics analyses by assigning individuals to the single cluster in which they exhibited the highest Q value.

The distance-based analysis was performed with Populations 1.2.30 (available at http://bioinformatics.org/~tryphon/populations/accessed on October 14, 2011) using the chord distance of Cavalli-Sforza & Edwards [66] that is considered to be appropriate for closely related groups [67]. The matrix of genetic distances between individuals was used to construct a phylogenetic network with the Neighbor-net (N-net) method [68] on SplitsTree 4.11.3 [69]. The factorial correspondence analysis (FCA) implemented on GENETIX software [70] which places strains in a three-dimensional space according to its relation of genotypes.

Descriptive statistics were calculated for the assigned population using the GDA software (available at http://hydrodictyon.eeb.uconn.edu/people/plewis/software.php accessed on October 14, 2011) to calculate the mean number of alleles (A), the observed (*H*_o_) and expected (*H*_e_) heterozygosity, and the inbreeding coefficient *F*_IS_. The significance of *F*_IS_ was tested in GENETIX software [70] with 1,000 bootstraps.

The *F*_ST_ estimates were calculated using MSA software [71] with significance (p) tested with a 1000 permutations.

## Results

### Descriptive analysis

In this study, we analyzed 162 *L. infantum* strains from 17 Brazilian states and 11 from one locality in Paraguay (Figure 1; Table S1). The MLMT profiles revealed the presence of 67 genotypes; among these, 45 were unique (Table S1). Unique genotypes were observed for strains that were obtained from the different hosts, including humans, dogs, fox and opossum. The genotype ‘TYPE 10’ was sampled 52 times from 14 Brazilian states and Paraguay, and it represented 30% of the strains, including both human and dog isolates (Table S1; Table 2). The second predominant genotype was ‘TYPE 22’, which was mainly composed of MS strains; it included 47% (17/36) of the strains from this state and only one strain from CE. Ten genotypes were shared between humans and dogs (Table 2; Table S1).

The maximum number of alleles per locus was 8 (mean 2.93; Table 1), and three loci were monomorphic (KLIST7031, Li71-5/2, CS20). With the exception of two loci (Lm2TG and Lm4TA), the most frequent allele represented more than 92% of the strains. The overall *H*_o_ for each locus ranged from 0 to 0.072 (mean = 0.013), and the values were always lower than *H*_e_, which ranged from 0 to 0.495 (mean = 0.102) (Table 1). Overall, the inbreeding coefficient was 0.869, and it ranged from −0.003 to 0.939 (Table 1). The *F*_IS_ for the two loci that presented negative values were not significantly different from zero (Table 1).

### Population structure analyses

The Δ*K* values indicated that the variation of the dataset is better explained when three populations are considered (Figure S1). The result of *K* = 3 indicates a population composition of 111 strains for POP1 (36 genotypes), 31 strains for POP2 (19 genotypes) and 31 strains for POP3 (12 genotypes) (Figure 2; Table S1). Analyses with another clustering method implemented on software BAPS 5 [72] showed the same clusters when K = 3, showing the consistence of the populations observed on our dataset (data not shown). The geographic distribution of each population was not homogeneous (Figure 1). POP1 was observed in 17 of the studied localities (16 Brazilian states and Paraguay), and in some areas, it was markedly predominant. Despite the large geographic distribution of POP2 (10 Brazilian states), it was predominant only in MT, where 85% of the strains belong to this population. Seventy-five percent of POP3 was composed of MS strains and the remaining 25% was composed of four other strains, each of which originated from different states.

Even though the Δ*K* values favored *K* = 3 (Figure S1), we also examined other *K* values (Figure S2). We found that at *K* = 2, the first cluster corresponded to POP1 but displayed a lower level of admixture. The second cluster was composed of POP2 strains, which included samples with high degrees of admixture, and strains from POP3, which had a low probability of admixture as observed for *K* = 3 (Figure S2). The POP3 strains formed a cluster at all of the *K* levels (from 3 to 8) (Figure S2). Ten strains of POP1 from Espírito Santo State also formed a cluster for *K*>4 (Q>95% for *K* = 3; Figure S2). Two additional clusters (with 14 and 16 strains) were observed for *K* values between five and eight, but they included strains from mixed geographic origins. For some foci (Amazonas [1], Minas Gerais [1], Pará [1] and Rio de Janeiro [3]), few strains were analyzed; although they did not present any specific characteristic, an increase in sample size may provide a more reliable indication of their relationship with other foci. These strains were within POP1, with the exception of that from Pará, which was grouped with the POP2 strains. Furthermore, the strain from Manaus, Amazonas State, displayed dubious anamnesis; this area is not considered to be endemic for VL, and the history of the patient could not be confirmed.

### Genetic diversity and phylogenetic analyses

Analyses of the genetic diversity within and between the populations were performed assuming the three populations obtained by the STRUCTURE analysis (POP1, POP2, and POP3; Figure 2; Figure S1). The *H*_o_ values per locus within the populations ranged from zero to 0.26, and *H*_e_ ranged from zero to 0.58. *H*_e_ was always higher than *H*_o_. The inbreeding coefficients were high in all but three of the loci (two from POP1 and one from POP3). The overall *F*_IS_ estimates for each population were 0.888, 0.676 and 0.603 for POP1, POP2 and POP3, respectively. The descriptive statistics of the three populations are shown in Table S2.

These three populations were supported by significant *F*_ST_ estimates (p<0.001). The pairwise comparisons (*F*_ST_) were higher when they involved POP3 (0.66 and 0.52 with POP1 and POP2, respectively) than between POP1 and POP2 (0.35).

The three-dimensional FCA confirmed the occurrence of the three populations predicted on STRUCTURE analysis and confirmed by *F*_ST_ estimates (Figure 3).

The splits graphic (N-net) essentially corroborates the other analyses. However, the presence of large splits indicates incongruences for the positions of some genotypes (Figure 4). POP1 formed a group of genotypes that were separated by short splits, and the other groups were more dispersed and divergent. Some POP2 genotypes were closely related to another population (POP1 or POP3) but do not constitute a distinct cluster. POP3 formed a cluster with simple splits among genotypes.

## Discussion

The VL dynamics have changed in recent decades; outbreaks have challenged the public health system, and new scenarios have intrigued researchers. Advances in typing techniques have gradually enhanced the knowledge of the natural history of this widespread disease and have contributed to epidemiologic studies. This study provides new insights into aspects of the population genetics of *L. infantum* from Brazil and its relation to the eco-epidemiologic aspects of this parasite species in some of its endemic areas in the Americas. The strains from six states were newly sampled with the MLMT approach, including Amazonas, Maranhão, Minas Gerais, São Paulo, Mato Grosso and strains from a recent focus of CVL in South Brazil (Rio Grande do Sul [RS] State).

The MLMT profiles of 173 *L. infantum* strains, which all belong to the same zymodeme, IOC/Z1, revealed 67 genotypes, which is the same proportion of polymorphic strains (39%) that was observed in a previous study that analyzed *L. infantum* strains from Central and South America [4]. In general, the *H*_o_ values were lower than the *H*_e_ values even within populations, which reflects a lack of heterozygotes, a result that is consistent with other microsatellite-based studies of *Leishmania* populations [4], [47], [48], [53], [54], [55], [59], [60]. Both estimates (*H*_e_ and *H*_o_) were lower than in the Old World MON-1 strains, as previously described; this corroborates the hypothesis of recent (less than 500 years) introduction of *L. infantum* in the Neotropics [4]. The lower genetic diversity of the *L. infantum* populations from the New World when compared to those from the Old World and the high proportion of the predominant allele for each locus support the hypothesis of the introduction and later expansion of a few clones in the Americas since the Colonial period; the expansion likely followed the paths of human migration from the endemic areas of Spain and Portugal [4], [62].

Interestingly, the *F*_IS_ estimates for the POP1 across two loci and one in POP3 were negative, but not statistically different from zero. The *F*_IS_ estimates for the other polymorphic loci were high, which indicates a depletion of heterozygotes and high endogamy, which is commonly observed in *Leishmania* species [4], [47], [48], [55], [59], [60], [73], [74]. It was previously suggested that the variation in the heterozygosity levels among the loci in *Leishmania* (*Viannia*) is associated with genetic exchange among parasites [75]. Another explanation for these high *F*_IS_ estimates may be the Wahlund effect, which is indicated by small consistent clusters that are observed in the hierarchical structure analysis (Figure S2).

The use of *H*_e_ as a measure of genetic diversity was proposed for sexual diploid organisms [76]. In theory, low genetic diversity is expected for strictly clonal organisms and, in absence of recombination, variation would only be generated through mutation events. In this case, the pairs of alleles accumulate mutations and become divergent which would lead to an excess of heterozygotes (Meselson effect) [77], [78]. Furthermore, if all of the individuals were heterozygotes, *F*_IS_ should be equal to −1 and any event of sexual reproduction could easily reduce heterozygosity [79], [80], [81], [82]. The high inbreeding estimates and the depletion of heterozygotes that were observed in Brazilian *L. infantum* populations suggest a predominantly clonal breeding system; however, even at very low frequencies, sexual reproduction should have occurred, as reported in other studies [4], [47], [48], [55], [60], [73], [74].

Furthermore, the presence of strains that contain traces of more than one population (Figure 2) also indicates the occurrence of gene flow because this mixed ancestry may be interpreted as either migrant descents or recombinants; both of these outcomes are the result of sexual breeding. Understanding the breeding structure of pathogens is not only an academic challenge and it has also epidemiological importance because it might affect the distribution of advantageous mutations (such as those conferring drug resistance) among strains or populations [83], [84]. A reproductive system where clonality is associated with low levels of sex could favor the rapid increase in frequency of hybrids with higher fitness [79]. This hypothesis however must be properly tested in *Leishmania*.

Although the molecular variability in our sampling was low, the clustering analysis detected the existence of three groups (POP1, POP2 and POP3; Figure 2) that were confirmed by FCA and distance-based methods (Figures 3 and 4) and were supported by *F* statistics. The signs of a population structure among the MON-1 strains from the New World that were analyzed by Kuhls et al. [4] were confirmed in the current work. The absence of statistical support for the New World genetic structure that was observed by Kuhls et al. [4] was likely a result of the lower sample size. POP1 and POP3 represent very similar populations of Sub-Pop1A-INF_NW_ and Sub-Pop1B-INF_NW_, respectively (Figure 2 in [4]), but they were supported by the *F*_ST_ estimate in the current study. However, the STRUCTURE analysis showed that many strains can be descendants of migrants, which may also lead to the occurrence of recombination as a consequence of sexual reproduction, as previously described [4], [59], [60], [85].

Interestingly, POP1 contains strains from 16 states and Paraguay, which accounted for 64% of the total sample set and 54% of the genotypes. POP1 includes few samples from MS and MT, where POP3 and POP2 were respectively predominant (Figure 1). This result may mean that, for some reason, the POP2 and POP3 genotypes possess higher fitness in Central West Brazil, perhaps due to adaptation to a different transmission cycle (see below).

The outbreaks of VL in MS and MT began in the late 1990s and early 2000s [35], [86]. A “historical series” has shown that in MS, the spread of VL epidemics occurs from west to east, following the construction of a road and gas pipeline [24]. POP3 is composed primarily of strains from MS, where VL has exhibited particularities such as the participation of *Lu. cruzi* on the transmission [34] and the urbanization of the disease [19], [25]. The capital of MS, Campo Grande, accounted for almost 50% of the cases in that state from 2001 to 2006, and these were primarily located in urban areas [86]. Urban cases were also predominant in MT [35]. Although MT and MS share some VL characteristics, they are influenced by different ecoregions. Most strains from MT (86%) were composed of POP2, whereas those in MS were predominantly composed of POP3. Surprisingly, POP2 was more closely related genetically to the widespread POP1 than to the geographic neighbor POP3, which can be seen from the STRUCTURE (Figure 2) and pairwise *F*_ST_ analyses.

Despite the presence of *Lu. longipalpis* in most of the foci from MS and MT, the sympatry with *Lu. cruzi* seems to be less prevalent in MS, where the latter species commonly accounts for a small percentage of collected sand flies or was simply not recorded in the same area [35], [36], [37], [86], [87]. The occurrence of *Lu. cruzi* is more relevant in a few areas, such as Corumbá (in western MS), one of the first localities on the west-east route of VL in MS. This suggests that this vector probably played an important role in shaping the genetic structure of *L. infantum*. Perhaps the POP3 genotypes were initially favored by the presence of *Lu. cruzi* in Corumbá, and then, following human interference, they dispersed through the eastern portion of the state. However, we did not include strains from any areas of high prevalence of *Lu. cruzi*, which prevents strong conclusions. The expansion of VL from west-east reached western SP [24] and has possibly influenced the distribution of the disease and *L. infantum* populations in this state. However, this possibility cannot be ruled out by our data because only four strains from Imbú das Artes (eastern SP) were analyzed.

Another way to explain the genetic structure of *L. infantum* in MS and MT is to assume that the recent spread of VL in Central West Brazil may be a consequence of the expansion from a few strains that were imported from other states, such as those from the Northeast Brazil. In fact, POP3 is predominantly observed in MS, but it is also present in four northeastern states in which VL is highly endemic. Furthermore, 70% of the POP3 MS strains were included in the same genotype ‘TYPE 22’ (Table S1), which was also recorded for one strain in Ceará State. We cannot exclude the possibility that this Ceará strain does not represent an autochthonous case. However, another explanation for this result is that this genotype entered MS from Northeast Brazil, where a highly successful adaptation facilitated a clonal expansion. It would be interesting to evaluate the possibility of an influence from Eastern Bolivia, although in that country, the only endemic area that has been reported is Los Yungas, La Paz, which does not seem to be connected to the Brazilian foci [88], [89].

The clustering of samples from Paraguay and RS in POP1 may indicate that the presence of VL in these areas is not a consequence of VL expansion from MS. It may be difficult to explain how Paraguay and RS are genetically closer to the Southeast and Northeast strains when considering a VL-distribution gap from southern SP to northern RS. Thus, it is conceivable that the dispersion of VL was not linear and may have been carried by asymptomatic hosts [90] from Southeast Brazil to some point in Northeastern Argentina or Southern Paraguay and then expanded to South Brazil. However, it is more likely that the few POP1 strains in MS may represent a dispersal link between this population and those areas.

The *L. infantum* RS samples that were studied here were isolated from dogs of three foci; two were isolated from areas that share geographic limits with Northeastern Argentina, where new cases were recently described [91] and from which they could be linked.

The recent expansion of VL to South Brazil underscores the importance of understanding the transmission cycle in outbreak areas, such as Argentina and Paraguay, and the genetic relationships between the parasites and the disease source. The *Leishmania* source should be investigated through molecular epidemiology tools, as was previously performed for *L. donovani* in Ethiopia, to determine the origin of outbreak strains [57].

In addition to the recent evidence regarding the introduction of *L. infantum* to the New World [4], [62], it is important to understand the dynamics and dispersion of AVL. The geographic population structure that is observed here does not discard the relationship with human migration, urban cycles and new endemic areas, and it also indicates the influence of vector species on the population structure of parasites. Further analyses are needed to elucidate how different vectors shape the variability in *L. infantum* populations. Moreover, an enhanced coverage and sampling area for some foci and micro-geographical studies could reveal interesting features of the natural history of this still-neglected disease.

## Supporting Information

Figure S1 **Delta** ***K*** **(Δ** ***K*** **) values of Evanno's method calculated with Structure Harvester v0.6.1.** The most probable *K* value *K* that explains the variation in the microsatellite data set was three.
(TIF)

Figure S2 **The distribution of Q values of** ***K*** **populations.** The distribution of the alignments for the Q values determined with the CLUMPP software for the chosen number of populations (2<*K*<8). The Δ*K* values indicated that the most probable number of populations is 3 (Figure S1). The squares show constant clusters with low degrees of admixture.
(TIF)

Table S1 **General information of the** ***Leishmania infantum*** **strains from Brazil and Paraguay analyzed in this study.**
(DOC)

Table S2 **Descriptive statistics of the** ***Leishmania infantum*** **MLMT profiles of populations from STRUCTURE analysis.**
(DOC)

## References

[1] WHO (2010). Control of the leishmaniases: report of a meeting of the WHO Expert Commitee on the Control of Leishmaniases. Geneva: World Health Organization..

[2] Lainson R, Shaw JJ (1987). The Leishmaniases in Biology and Medicine: Evolution, classification and geographical distribution; Peters W, Killick-Kendrick R, editors..

[3] Rioux JA, Lanotte G, Serres E, Pratlong F, Bastien P (1990). Taxonomy of *Leishmania*. Use of isoenzymes. Suggestions for a new classification.. Ann Parasitol Hum Comp.

[4] Kuhls K, Alam MZ, Cupolillo E, Ferreira GE, Mauricio IL (2011). Comparative microsatellite typing of New World *Leishmania infantum* reveals low heterogeneity among populations and its recent Old World origin.. PLoS Negl Trop Dis.

[5] Cunha AMU (1938). Infecções experimentaes na Leishmaniose visceral americana.. Memórias do Instituto Oswaldo Cruz.

[6] Killick-Kendrick R, Molyneux DH, Rioux JA, Lanotte G, Leaney AJ (1980). Possible origins of *Leishmania chagasi*.. Ann Trop Med Parasitol.

[7] Momen H, Grimaldi Junior G, Deane LM (1987). *Leishmania infantum*, the aetiological agent of American visceral leishmaniasis (AVL)?. Mem Inst Oswaldo Cruz.

[8] Momen H, Pacheco RS, Cupolillo E, Grimaldi Junior G (1993). Molecular evidence for the importation of Old World *Leishmania* into the Americas.. Biol Res.

[9] Shaw JJ (1994). Taxonomy of the genus *Leishmania*: present and future trends and their implications.. Mem Inst Oswaldo Cruz.

[10] Grimaldi G, Tesh RB, McMahon-Pratt D (1989). A review of the geographic distribution and epidemiology of leishmaniasis in the New World.. Am J Trop Med Hyg.

[11] Romero GA, Boelaert M (2010). Control of visceral leishmaniasis in Latin America – a systematic review.. PLoS Negl Trop Dis.

[12] Rosypal AC, Troy GC, Zajac AM, Duncan RB, Waki K (2003). Emergence of zoonotic canine leishmaniasis in the United States: isolation and immunohistochemical detection of *Leishmania infantum* from foxhounds from Virginia..

[13] Werneck GL (2010). Geographic spread of visceral leishmaniasis in Brazil.. Cad Saude Publica.

[14] Alexander B, de Carvalho RL, McCallum H, Pereira MH (2002). Role of the domestic chicken (*Gallus gallus*) in the epidemiology of urban visceral leishmaniasis in Brazil.. Emerg Infect Dis.

[15] Jeronimo SM, Oliveira RM, Mackay S, Costa RM, Sweet J (1994). An urban outbreak of visceral leishmaniasis in Natal, Brazil.. Trans R Soc Trop Med Hyg.

[16] Maia-Elkhoury AN, Alves WA, Sousa-Gomes ML, Sena JM, Luna EA (2008). Visceral leishmaniasis in Brazil: trends and challenges.. Cad Saude Publica.

[17] Oliveira AG, Galati EAB, Oliveira O, Oliveira GR, Espindola IAC (2006). Abundance of *Lutzomyia longipalpis* (Diptera: Psychodidae: Phlebotominae) and urban transmission of visceral leishmaniasis in Campo Grande, state of Mato Grosso do Sul, Brazil.. Memórias do Instituto Oswaldo Cruz.

[18] Rangel EF, Vilela ML (2008). *Lutzomyia longipalpis* (Diptera, Psychodidae, Phlebotominae) and urbanization of visceral leishmaniasis in Brazil.. Cad Saude Publica.

[19] Werneck GL (2008). Forum: geographic spread and urbanization of visceral leishmaniasis in Brazil. Introduction.. Cad Saude Publica.

[20] Brazil RP, De Almeida DC, Brazil BG, Mamede SM (1991). Chicken house as a resting site of sandflies in Rio de Janeiro, Brazil..

[21] Deane LM, Deane MP (1954). Dogs naturally infected by *Leishmania donovani* in Ceara.. Hospital (Rio J).

[22] Dias FOP, Lorosa ES, Rebêlo JMM (2003). Fonte alimentar sangüínea e a peridomiciliação de *Lutzomyia longipalpis* (Lutz & Neiva, 1912) (Psychodidae, Phlebotominae).. Cadernos de Saúde Pública.

[23] Palatnik-de-Sousa CB, dos Santos WR, Franca-Silva JC, da Costa RT, Reis AB (2001). Impact of canine control on the epidemiology of canine and human visceral leishmaniasis in Brazil.. Am J Trop Med Hyg.

[24] Antonialli SAC, Torres TG, Paranhos Filho AC, Tolezano JE (2007). Spatial analysis of American Visceral Leishmaniasis in Mato Grosso do Sul State, Central Brazil.. J Infect.

[25] Oliveira AL, Paniago AM, Dorval ME, Oshiro ET, Leal CR (2006). Emergent outbreak of visceral leishmaniasis in Mato Grosso do Sul State.. Rev Soc Bras Med Trop.

[26] Oliveira CD, Assuncao RM, Reis IA, Proietti FA (2001). Spatial distribution of human and canine visceral leishmaniasis in Belo Horizonte, Minas Gerais State, Brasil, 1994–1997.. Cad Saude Publica.

[27] Silva ES, Gontijo CM, Pacheco RS, Fiuza VO, Brazil RP (2001). Visceral leishmaniasis in the Metropolitan Region of Belo Horizonte, State of Minas Gerais, Brazil.. Mem Inst Oswaldo Cruz.

[28] Souza GD, Santos E, Andrade Filho JD (2009). The first report of the main vector of visceral leishmaniasis in America, *Lutzomyia longipalpis* (Lutz & Neiva) (Diptera: Psychodidae: Phlebotominae), in the state of Rio Grande do Sul, Brazil.. Mem Inst Oswaldo Cruz.

[29] Lainson R, Rangel EF (2005). *Lutzomyia longipalpis* and the eco-epidemiology of American visceral leishmaniasis, with particular reference to Brazil: a review.. Mem Inst Oswaldo Cruz.

[30] Travi BL, Velez ID, Brutus L, Segura I, Jaramillo C (1990). *Lutzomyia evansi*, an alternate vector of *Leishmania chagasi* in a Colombian focus of visceral leishmaniasis.. Trans R Soc Trop Med Hyg.

[31] de Carvalho MR, Valenca HF, da Silva FJ, de Pita-Pereira D, de Araujo Pereira T (2010). Natural *Leishmania infantum* infection in *Migonemyia migonei* (França, 1920) (Diptera:Psychodidae:Phlebotominae) the putative vector of visceral leishmaniasis in Pernambuco State, Brazil.. Acta Trop.

[32] de Pita-Pereira D, Cardoso MA, Alves CR, Brazil RP, Britto C (2008). Detection of natural infection in *Lutzomyia cruzi* and *Lutzomyia forattinii* (Diptera: Psychodidae: Phlebotominae) by *Leishmania infantum chagasi* in an endemic area of visceral leishmaniasis in Brazil using a PCR multiplex assay.. Acta Trop.

[33] Missawa NA, Veloso MA, Maciel GB, Michalsky EM, Dias ES (2011). Evidence of transmission of visceral leishmaniasis by *Lutzomyia cruzi* in the municipality of Jaciara, State of Mato Grosso, Brazil.. Rev Soc Bras Med Trop.

[34] dos Santos SO, Arias J, Ribeiro AA, de Paiva Hoffmann M, de Freitas RA (1998). Incrimination of *Lutzomyia cruzi* as a vector of American visceral leishmaniasis.. Med Vet Entomol.

[35] Mestre GL, Fontes CJ (2007). The spread of the visceral leishmaniasis epidemic in the State of Mato Grosso, 1998–2005.. Rev Soc Bras Med Trop.

[36] Missawa NA, Lima GB (2006). Spatial distribution of *Lutzomyia longipalpis* (Lutz & Neiva, 1912) and *Lutzomyia cruzi* (Mangabeira, 1938) in the State of Mato Grosso.. Rev Soc Bras Med Trop.

[37] Oliveira AG, Andrade Filho JD, Falcão AL, Brazil RP (2003). Estudo de flebotomíneos (Diptera, Psychodidae, Phlebotominae) na zona urbana da cidade de Campo Grande, Mato Grosso do Sul, Brasil, 1999–2000.. Cadernos de Saúde Pública.

[38] Ribeiro AL, Missawa NA, Zeilhofer P (2007). Distribution of phlebotomine sandflies (Diptera: Psychodidae) of medical importance in Mato Grosso State, Brazil.. Rev Inst Med Trop Sao Paulo.

[39] Araki AS, Vigoder FM, Bauzer LG, Ferreira GE, Souza NA (2009). Molecular and behavioral differentiation among Brazilian populations of *Lutzomyia longipalpis* (Diptera: Psychodidae: Phlebotominae).. PLoS Negl Trop Dis.

[40] Vigoder FM, Araki AS, Bauzer LG, Souza NA, Brazil RP (2010). Lovesongs and *period* gene polymorphisms indicate *Lutzomyia cruzi* (Mangabeira, 1938) as a sibling species of the *Lutzomyia longipalpis* (Lutz and Neiva, 1912) complex.. Infect Genet Evol.

[41] Watts PC, Hamilton JG, Ward RD, Noyes HA, Souza NA (2005). Male sex pheromones and the phylogeographic structure of the *Lutzomyia longipalpis* species complex (Diptera: Psychodidae) from Brazil and Venezuela.. Am J Trop Med Hyg.

[42] Banuls AL, Hide M, Prugnolle F (2007). *Leishmania* and the leishmaniases: a parasite genetic update and advances in taxonomy, epidemiology and pathogenicity in humans.. Adv Parasitol.

[43] Ajzenberg D, Collinet F, Mercier A, Vignoles P, Darde ML (2010). Genotyping of *Toxoplasma gondii* isolates with 15 microsatellite markers in a single multiplex PCR assay.. J Clin Microbiol.

[44] Bruce MC, Macheso A, McConnachie A, Molyneux ME (2011). Comparative population structure of *Plasmodium malariae* and *Plasmodium falciparum* under different transmission settings in Malawi.. Malar J.

[45] Conrad M, Zubacova Z, Dunn LA, Upcroft J, Sullivan SA (2011). Microsatellite polymorphism in the sexually transmitted human pathogen *Trichomonas vaginalis* indicates a genetically diverse parasite.. Mol Biochem Parasitol.

[46] Hennequin C, Thierry A, Richard GF, Lecointre G, Nguyen HV (2001). Microsatellite typing as a new tool for identification of *Saccharomyces cerevisiae* strains.. J Clin Microbiol.

[47] Al-Jawabreh A, Diezmann S, Muller M, Wirth T, Schnur LF (2008). Identification of geographically distributed sub-populations of *Leishmania* (*Leishmania*) major by microsatellite analysis.. BMC Evol Biol.

[48] Oddone R, Schweynoch C, Schonian G, Sousa C S, Cupolillo E (2009). Development of a multilocus microsatellite typing approach for discriminating strains of *Leishmania* (*Viannia*) species.. J Clin Microbiol.

[49] Reale S, Lupo T, Migliazzo A, Di Mauro C, Cipri V (2010). Multilocus microsatellite polymorphism analysis to characterize *Leishmania infantum* strains isolated in Sicily.. Transbound Emerg Dis.

[50] Rougeron V, Waleckx E, Hide M, T DEM, Arevalo J (2008). PERMANENT GENETIC RESOURCES: A set of 12 microsatellite loci for genetic studies of *Leishmania braziliensis*.. Mol Ecol Resour.

[51] Russell R, Iribar MP, Lambson B, Brewster S, Blackwell JM (1999). Intra and inter-specific microsatellite variation in the *Leishmania* subgenus *Viannia*.. Mol Biochem Parasitol.

[52] Ochsenreither S, Kuhls K, Schaar M, Presber W, Schonian G (2006). Multilocus microsatellite typing as a new tool for discrimination of *Leishmania infantum* MON-1 strains.. J Clin Microbiol.

[53] Alam MZ, Kovalenko DA, Kuhls K, Nasyrova RM, Ponomareva VI (2009). Identification of the agent causing visceral leishmaniasis in Uzbeki and Tajiki foci by analysing parasite DNA extracted from patients' Giemsa-stained tissue preparations.. Parasitology.

[54] Alam MZ, Kuhls K, Schweynoch C, Sundar S, Rijal S (2009). Multilocus microsatellite typing (MLMT) reveals genetic homogeneity of *Leishmania donovani* strains in the Indian subcontinent.. Infect Genet Evol.

[55] Amro A, Schonian G, Al-Sharabati MB, Azmi K, Nasereddin A (2009). Population genetics of *Leishmania infantum* in Israel and the Palestinian Authority through microsatellite analysis.. Microbes Infect.

[56] Chargui N, Amro A, Haouas N, Schonian G, Babba H (2009). Population structure of Tunisian *Leishmania infantum* and evidence for the existence of hybrids and gene flow between genetically different populations.. Int J Parasitol.

[57] Gelanew T, Cruz I, Kuhls K, Alvar J, Canavate C (2011). Multilocus microsatellite typing revealed high genetic variability of *Leishmania donovani* strains isolated during and after a Kala-azar epidemic in Libo Kemkem district, northwest Ethiopia.. Microbes Infect.

[58] Gelanew T, Kuhls K, Hurissa Z, Weldegebreal T, Hailu W (2010). Inference of population structure of *Leishmania donovani* strains isolated from different Ethiopian visceral leishmaniasis endemic areas.. PLoS Negl Trop Dis.

[59] Kuhls K, Chicharro C, Canavate C, Cortes S, Campino L (2008). Differentiation and gene flow among European populations of *Leishmania infantum* MON-1.. PLoS Negl Trop Dis.

[60] Kuhls K, Keilonat L, Ochsenreither S, Schaar M, Schweynoch C (2007). Multilocus microsatellite typing (MLMT) reveals genetically isolated populations between and within the main endemic regions of visceral leishmaniasis.. Microbes Infect.

[61] Seridi N, Amro A, Kuhls K, Belkaid M, Zidane C (2008). Genetic polymorphism of Algerian *Leishmania infantum* strains revealed by multilocus microsatellite analysis.. Microbes Infect.

[62] Leblois R, Kuhls K, Francois O, Schonian G, Wirth T (2011). Guns, germs and dogs: On the origin of *Leishmania chagasi*.. Infect Genet Evol.

[63] Pritchard JK, Stephens M, Donnelly P (2000). Inference of population structure using multilocus genotype data.. Genetics.

[64] Evanno G, Regnaut S, Goudet J (2005). Detecting the number of clusters of individuals using the software STRUCTURE: a simulation study.. Mol Ecol.

[65] Jakobsson M, Rosenberg NA (2007). CLUMPP: a cluster matching and permutation program for dealing with label switching and multimodality in analysis of population structure.. Bioinformatics.

[66] Cavalli-Sforza LL, Edwards AW (1967). Phylogenetic analysis. Models and estimation procedures.. Am J Hum Genet.

[67] Goldstein DB, Pollock DD (1997). Launching microsatellites: a review of mutation processes and methods of phylogenetic interference.. J Hered.

[68] Bryant D, Moulton V (2004). Neighbor-net: an agglomerative method for the construction of phylogenetic networks.. Mol Biol Evol.

[69] Huson DH, Bryant D (2006). Application of phylogenetic networks in evolutionary studies.. Mol Biol Evol.

[70] Belkhir K, Borsa P, Chikhi L, Raufaste N, Bonhomme F (1996). GENETIX 4.05, logiciel sous Windows TM pour la génétique des populations..

[71] Dieringer D, Schlötterer C (2003). Microsatellite analyser (MSA): a platform independent analysis tool for large microsatellite data sets.. Molecular Ecology Notes.

[72] Corander J, Marttinen P, Siren J, Tang J (2008). Enhanced Bayesian modelling in BAPS software for learning genetic structures of populations.. BMC Bioinformatics.

[73] Rougeron V, De Meeus T, Hide M, Waleckx E, Bermudez H (2009). Extreme inbreeding in *Leishmania braziliensis*.. Proc Natl Acad Sci U S A.

[74] Rougeron V, De Meeus T, Hide M, Waleckx E, Dereure J (2010). A battery of 12 microsatellite markers for genetic analysis of the *Leishmania* (*Viannia*) *guyanensis* complex.. Parasitology.

[75] Nolder D, Roncal N, Davies CR, Llanos-Cuentas A, Miles MA (2007). Multiple hybrid genotypes of *Leishmania* (*Viannia*) in a focus of mucocutaneous Leishmaniasis.. Am J Trop Med Hyg.

[76] Nei M, Kumar S (2000). Molecular evolution and phylogenetics New York: Oxford University Press.

[77] Butlin R (2002). Evolution of sex: The costs and benefits of sex: new insights from old asexual lineages.. Nat Rev Genet.

[78] Pouchkina-Stantcheva NN, McGee BM, Boschetti C, Tolleter D, Chakrabortee S (2007). Functional divergence of former alleles in an ancient asexual invertebrate.. Science.

[79] Balloux F, Lehmann L, de Meeus T (2003). The population genetics of clonal and partially clonal diploids.. Genetics.

[80] de Meeus T, Balloux F (2005). *F*-statistics of clonal diploids structured in numerous demes.. Mol Ecol.

[81] de Meeus T, Lehmann L, Balloux F (2006). Molecular epidemiology of clonal diploids: a quick overview and a short DIY (do it yourself) notice.. Infect Genet Evol.

[82] Salim B, de Meeus T, Bakheit MA, Kamau J, Nakamura I (2011). Population genetics of *Trypanosoma evansi* from camel in the Sudan.. PLoS Negl Trop Dis.

[83] Tibayrenc M (1997). Are *Candida albicans* natural populations subdivided?. Trends Microbiol 5: 253–254; discussion.

[84] Tibayrenc M, Kjellberg F, Arnaud J, Oury B, Breniere SF (1991). Are eukaryotic microorganisms clonal or sexual? A population genetics vantage.. Proc Natl Acad Sci U S A.

[85] Rougeron V, Banuls AL, Carme B, Simon S, Couppie P (2011). Reproductive strategies and population structure in *Leishmania*: substantial amount of sex in *Leishmania Viannia guyanensis*.. Mol Ecol.

[86] Botelho AC, Natal D (2009). First epidemiological description of visceral leishmaniasis in Campo Grande, State of Mato Grosso do Sul.. Rev Soc Bras Med Trop.

[87] Nascimento JC, Paiva BR, Malafronte RS, Fernandes WD, Galati EAB (2007). Natural infection of phlebotomines (Diptera: Psychodidae) in a visceral-leishmaniasis focus in Mato Grosso do Sul, Brazil.. Revista do Instituto de Medicina Tropical de São Paulo.

[88] Desjeux P, Aranda E, Aliaga O, Mollinedo S (1983). Human visceral leishmaniasis in Bolivia: first proven autochthonous case from ‘Los Yungas’.. Trans R Soc Trop Med Hyg.

[89] Garcia AL, Parrado R, Rojas E, Delgado R, Dujardin JC (2009). Leishmaniases in Bolivia: comprehensive review and current status.. Am J Trop Med Hyg.

[90] Michel G, Pomares C, Ferrua B, Marty P (2011). Importance of worldwide asymptomatic carriers of *Leishmania infantum* (*L. chagasi*) in human.. Acta Trop.

[91] Salomon O, Sinagra A, Nevot M, Barberian G, Paulin P (2008). First visceral leishmaniasis focus in Argentina.. Mem Inst Oswaldo Cruz.

