# Supplementary figures and images for: The Genetic Structure of Leishmania infantum Populations in Brazil and Its Possible Association with the Transmission Cycle of Visceral Leishmaniasis
Source: PLoS One. 2012 May 11;7(5):e36242. doi: 10.1371/journal.pone.0036242 (PMC3350531; doi:10.1371/journal.pone.0036242)

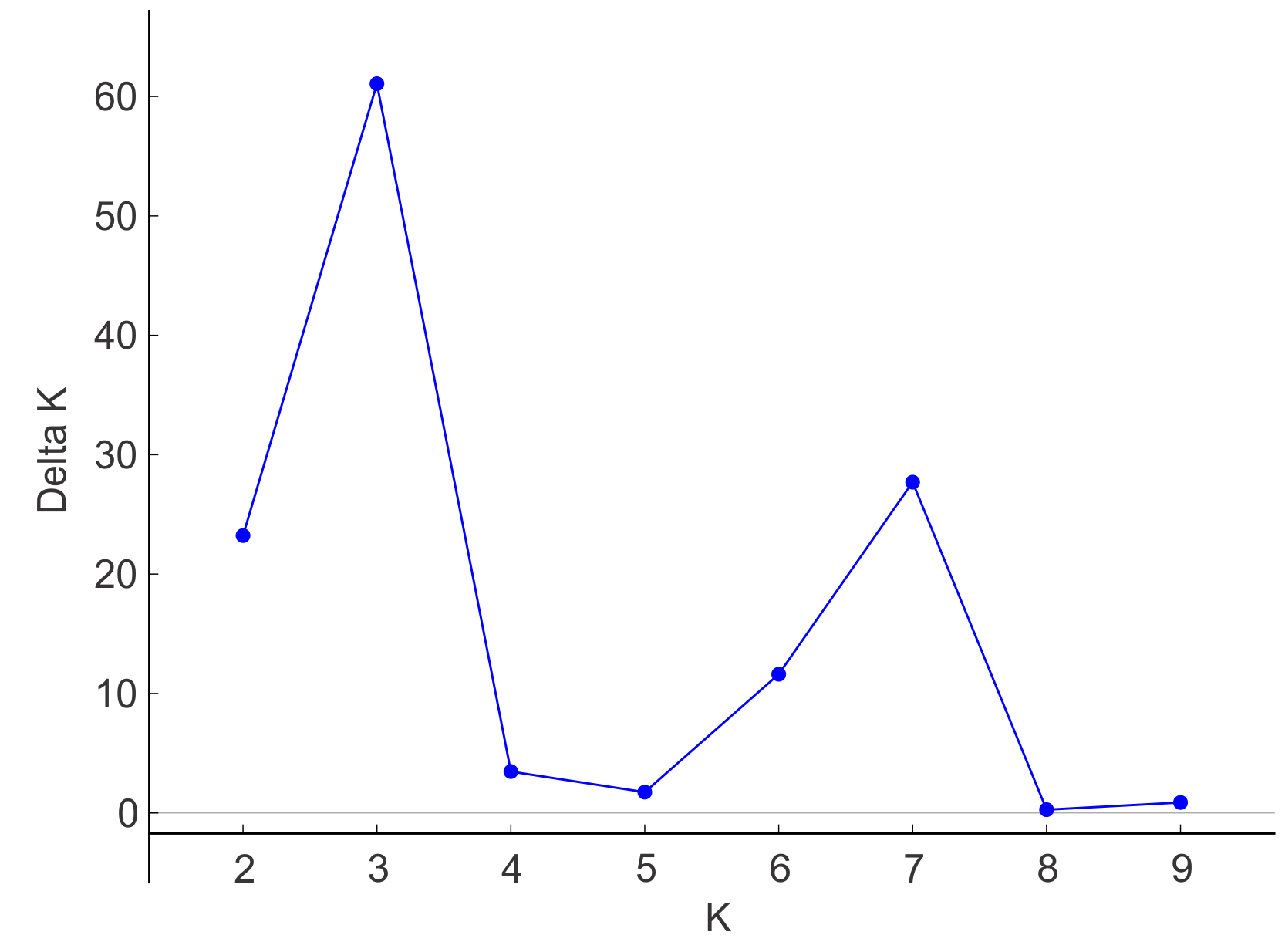

Supplement: Figure S1 — Delta K (Δ K ) values of Evanno's method calculated with Structure Harvester v0.6.1. The most probable K value K that explains the variation in the microsatellite data set was three. (TIF) [file pone.0036242.s001.tif]

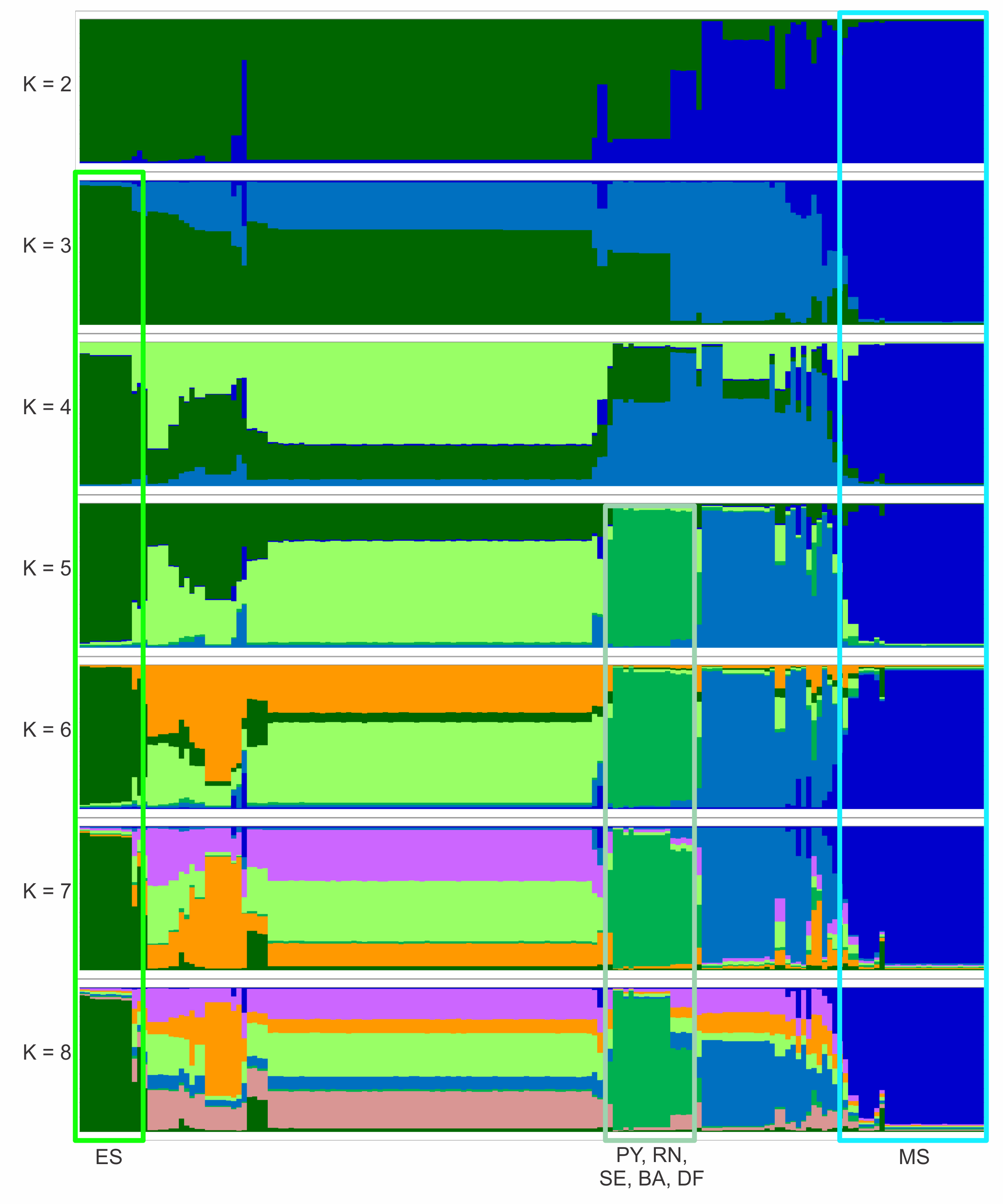

Supplement: Figure S2 — The distribution of Q values of K populations. The distribution of the alignments for the Q values determined with the CLUMPP software for the chosen number of populations (2<K<8). The ΔK values indicated that the most probable number of populations is 3 (Figure S1). The squares show constant clusters with low degrees of admixture. (TIF) [file pone.0036242.s002.tif]
