# Supplementary material for: The Genetic Structure of Leishmania infantum Populations in Brazil and Its Possible Association with the Transmission Cycle of Visceral Leishmaniasis
Source: PLoS One. 2012 May 11;7(5):e36242. doi: 10.1371/journal.pone.0036242 (PMC3350531; doi:10.1371/journal.pone.0036242)
Supplement: Table S1 — General information of the Leishmania infantum strains from Brazil and Paraguay analyzed in this study. (DOC) [file pone.0036242.s003.doc]

**Table S1. General information of the *Leishmania infantum*** strains from Brazil and Paraguay analyzed in this study.

| Pop ID | SplitsTree genotype | IOC/L | International Code | State Code | Municipality |
| --- | --- | --- | --- | --- | --- |
| 1 | 'TYPE1' | 2926 | MCAN/BR/2006/CP-67 | BR-ES | Pancas |
| 1 | 'TYPE1' | 3022 | MCAN/BR/2007/CP-65 | BR-ES | Pancas |
| 1 | 'TYPE1' | 3023 | MCAN/BR/2007/CP-14 | BR-ES | Pancas |
| 1 | 'TYPE1' | 3024 | MCAN/BR/2007/CP-21 | BR-ES | Pancas |
| 1 | 'TYPE1' | 3026 | MCAN/BR/2007/CP-53 | BR-ES | Pancas |
| 1 | 'TYPE1' | 3066 | MCAN/BR/2008/CP-64 | BR-ES | Pancas |
| 1 | 'TYPE2' | 2507 | MCAN/BR/2002/CP-TARZAN | BR-ES | Pancas |
| 1 | 'TYPE2' | 2925 | MCAN/BR/2006/CBG05 | BR-ES | Baixo Guandú |
| 1 | 'TYPE3' | 3029 | MCAN/BR/2007/CP-60 | BR-ES | Pancas |
| 1 | 'TYPE3' | 3034 | MHOM/PY/2007/AS-8 | PY-ASU | Asunción |
| 1 | 'TYPE4' | 579 | MHOM/BR/1974/PP75 | BR-BA | Ituaçú |
| 1 | 'TYPE4' | 2906 | MHOM/BR/2002/LPC-RPV | BR-MG | Belo Horizonte |
| 1 | 'TYPE5' | 2788 | MHOM/BR/2005/DRD | BR-ES | Rio Novo do Sul |
| 1 | 'TYPE5' | 2789*^§^* | MHOM/BR/2005/HRNS-1 | BR-ES | Rio Novo do Sul |
| 1 | 'TYPE6' | 3245 | MCAN/BR/2011/CLV7 | BR-SP | Imbú das Artes |
| 1 | 'TYPE6' | 3247 | MCAN/BR/2011/CLV18 | BR-SP | Imbú das Artes |
| 1 | 'TYPE7' | 3104 | MCAN/PY/2009/LVH-HIV2 | PY-ASU | Asunción |
| 1 | 'TYPE7' | 3115 | MHOM/BR/2009/HU-UFS10 | BR-SE | Aracaju |
| 1 | 'TYPE8' | 3193 | MCAN/BR/2010/LEONA | BR-RS | Uruguaiana |
| 1 | 'TYPE8' | 3198 | MCAN/BR/2010/LAIKA | BR-RS | Uruguaiana |
| 1 | 'TYPE9' | 3056 | MHOM/BR/2008/HUB-124832-MO | BR-DF | Brasília |
| 1 | 'TYPE9' | 3107 | MHOM/BR/2009/HU-UFS02 | BR-SE | Aracaju |
| 1 | 'TYPE10' | 636 | MHOM/BR/1985/NJ | BR-BA | Jacobina |
| 1 | 'TYPE10' | 641 | MHOM/BR/1985/SJS | BR-BA | Caen |
| 1 | 'TYPE10' | 1849*^§^* | MCAN/BR/1991/1373BAJE | BR-BA | Jequié |
| 1 | 'TYPE10' | 1850 | MCAN/BR/1991/1486BAJE | BR-BA | Jequié |
| 1 | 'TYPE10' | 2828 | MCAN/BR/2004/BUSH | BR-BA | Camaçari |
| 1 | 'TYPE10' | 329 | MCAN/BR/1984/17.206 | BR-CE | Canindé |
| 1 | 'TYPE10' | 606 | MCAN/BR/1984/CCC17.482 | BR-CE | Caucaia |
| 1 | 'TYPE10' | 665*^§^* | MCAN/BR/1986/CCC17.580 | BR-CE | São Gonçalo do Amarante |
| 1 | 'TYPE10' | 781 | MHOM/BR/1986/H-64(1P) | BR-CE | Trairi |
| 1 | 'TYPE10' | 902*^§^* | MCAN/BR/1987/CCC18.406 | BR-CE | Aquiraz |
| 1 | 'TYPE10' | 2920 | MCAN/BR/2006/NMT-DF007MO | BR-DF | Brasília |
| 1 | 'TYPE10' | 3055 | MHOM/BR/2008/HUB-347983-AE | BR-DF | Brasília |
| 1 | 'TYPE10' | 3057 | MHOM/BR/2008/HUB-466924-MO | BR-DF | Brasília |
| 1 | 'TYPE10' | 3058 | MHOM/BR/2008/HUB-064479-MO | BR-DF | Brasília |
| 1 | 'TYPE10' | 3079 | MHOM/BR/2006/HUB-403845-MOR2 | BR-DF | Brasília |
| 1 | 'TYPE10' | 3080 | MHOM/BR/2006/HUB-402498-MOR6 | BR-DF | Brasília |
| 1 | 'TYPE10' | 956*^§^* | MHOM/BR/1987/HBG-2 | BR-ES | Baixo Guandú |
| 1 | 'TYPE10' | 957 | MHOM/BR/1987/HCO-1 | BR-ES | Colatina |
| 1 | 'TYPE10' | 963 | MHOM/BR/1987/HIT-1 | BR-ES | Itaguaçu |
| 1 | 'TYPE10' | 3091 | MHOM/BR/2009/LVRS | BR-MA | São Luís |
| 1 | 'TYPE10' | 3092 | MHOM/BR/2009/LCS | BR-MA | São Luís |
| 1 | 'TYPE10' | 2776 | MHOM/BR/2005/Phufms-50 | BR-MS | Bela Vista |
| 1 | 'TYPE10' | 2986*^§^* | MHOM/BR/2007/JFVL | BR-MS | Campo Grande |
| 1 | 'TYPE10' | 3033 | MCAN/BR/2007/CG-1 | BR-MS | Campo Grande |
| 1 | 'TYPE10' | 2647*^§^* | MHOM/BR/2003/ACS | BR-PE | Recife |
| 1 | 'TYPE10' | 2763*^§^* | MCAN/BR/2005/FACHIDE | BR-PE | Recife |
| 1 | 'TYPE10' | 2933*^§^* | MCAN/BR/2006/MAIKE | BR-PE | Recife |
| 1 | 'TYPE10' | 3053 | MHOM/BR/2008/RJS | BR-PE | Recife |
| 1 | 'TYPE10' | 2949 | MCAN/BR/2004/LIBPI-18 | BR-PI | Teresina |
| 1 | 'TYPE10' | 3019 | MCAN/BR/2007/LIBPI-51 | BR-PI | Teresina |
| 1 | 'TYPE10' | 3097 | MCAN/PY/2009/EG20 | PY-ASU | Asunción |
| 1 | 'TYPE10' | 3099 | MCAN/PY/2009/EG35 | PY-ASU | Asunción |
| 1 | 'TYPE10' | 3100 | MCAN/PY/2009/EG36 | PY-ASU | Asunción |
| 1 | 'TYPE10' | 3101 | MCAN/PY/2009/LVH338 | PY-ASU | Asunción |
| 1 | 'TYPE10' | 3102 | MCAN/PY/2009/LVH560 | PY-ASU | Asunción |
| 1 | 'TYPE10' | 3103 | MCAN/PY/2009/LVH441 | PY-ASU | Asunción |
| 1 | 'TYPE10' | 45*^§^* | MCAN/BR/1980/CR3 | BR-RJ | Rio de Janeiro |
| 1 | 'TYPE10' | 3015*^§^* | MHOM/BR/2007/WC | BR-RJ | Rio de Janeiro |
| 1 | 'TYPE10' | 3020 | MHOM/BR/2007/JFF | BR-RJ | Cabo Frio |
| 1 | 'TYPE10' | 3054 | MHOM/BR/2008/3310 | BR-RN | Natal |
| 1 | 'TYPE10' | 3176 | MCAN/BR/2010/PV63 | BR-RN | Natal |
| 1 | 'TYPE10' | 3182 | MCAN/BR/2010/PV69 | BR-RN | Natal |
| 1 | 'TYPE10' | 3185 | MCAN/BR/2010/PV73 | BR-RN | Natal |
| 1 | 'TYPE10' | 3188 | MHOM/BR/2010/1070 | BR-RN | Natal |
| 1 | 'TYPE10' | 3083 | MCAN/BR/2009/CAO3-BAÇO | BR-RS | São Borja |
| 1 | 'TYPE10' | 3084 | MCAN/BR/2009/CAO3-PELE | BR-RS | São Borja |
| 1 | 'TYPE10' | 3191 | MCAN/BR/2010/DIANA I | BR-RS | Uruguaiana |
| 1 | 'TYPE10' | 3108 | MHOM/BR/2009/HU-UFS03 | BR-SE | Aracaju |
| 1 | 'TYPE10' | 3113 | MHOM/BR/2009/HU-UFS08 | BR-SE | Aracaju |
| 1 | 'TYPE10' | 3116 | MHOM/BR/2009/HU-UFS11 | BR-SE | Aracaju |
| 1 | 'TYPE10' | 3246 | MCAN/BR/2011/CLV8 | BR-SP | Imbú das Artes |
| 1 | 'TYPE10' | 3279 | MCAN/BR/2011/CLV1 | BR-SP | Imbú das Artes |
| 1 | 'TYPE11' | 2786 | MCAN/BR/2005/CP-55 | BR-ES | Pancas |
| 1 | 'TYPE11' | 3196 | MCAN/BR/2010/LUNA II | BR-RS | Uruguaiana |
| 1 | 'TYPE12' | 3105 | MHOM/BR/2009/ANC | BR-MA | São Luís |
| 1 | 'TYPE12' | 3117 | MHOM/BR/2009/VV | BR-MA | São Luís |
| 1 | 'TYPE12' | 3030 | MCAN/BR/2007/LIBPI-49 | BR-PI | Teresina |
| 1 | 'TYPE12' | 3031 | MCAN/BR/2007/LIBPI-54 | BR-PI | Teresina |
| 1 | 'TYPE13' | 3094 | MCAN/PY/2009/EG10 | PY-ASU | Asunción |
| 1 | 'TYPE13' | 3096 | MCAN/PY/2009/EG17 | PY-ASU | Asunción |
| 1 | 'TYPE13' | 3098 | MCAN/PY/2009/EG22 | PY-ASU | Asunción |
| 1 | 'TYPE14' | 3217 | MCAN/BR/2010/256 I | BR-MT | Cuiabá |
| 1 | 'TYPE14' | 3050 | MHOM/BR/2008/RN-04 | BR-RN | Natal |
| 1 | 'TYPE14' | 3178 | MCAN/BR/2010/PV65 | BR-RN | Natal |
| 1 | 'TYPE14' | 3183 | MCAN/BR/2010/PV71 | BR-RN | Natal |
| 1 | 'TYPE14' | 3184 | MCAN/BR/2010/PV72 | BR-RN | Natal |
| 1 | 'TYPE14' | 3186 | MCAN/BR/2010/PV74 | BR-RN | Natal |
| 2 | 'TYPE15' | 3021 | MCAN/BR/2007/CBG-12 | BR-ES | Baixo Guandú |
| 2 | 'TYPE15' | 2579 | MHOM/BR/2003/WAZ | BR-MS | São Gabriel do Oeste |
| 2 | 'TYPE16' | 2688 | MCAN/BR/2002/JACK CUSTEAU | BR-MS | Campo Grande |
| 2 | 'TYPE16' | 2985*^§^* | MHOM/BR/2007/JVF | BR-MS | Campo Grande |
| 2 | 'TYPE16' | 3110 | MHOM/BR/2009/HU-UFS05 | BR-SE | Aracaju |
| 2 | 'TYPE16' | 3112 | MHOM/BR/2009/HU-UFS07 | BR-SE | Aracaju |
| 2 | 'TYPE17' | 2593 | MHOM/BR/2003/BSB | BR-MS | Ribas do Rio Pardo |
| 2 | 'TYPE17' | 2665*^§^* | MCAN/BR/2002/LVV-136 | BR-MS | Campo Grande |
| 2 | 'TYPE17' | 3202 | MCAN/BR/2010/AKIRA I | BR-MT | Cuiabá |
| 2 | 'TYPE17' | 3208 | MCAN/BR/2010/ZEUS | BR-MT | Cuiabá |
| 2 | 'TYPE17' | 3226 | MCAN/BR/2010/CHITARA II | BR-MT | Cuiabá |
| 2 | 'TYPE17' | 3227 | MCAN/BR/2010/MAGRÃO | BR-MT | Cuiabá |
| 2 | 'TYPE18' | 3076 | MHOM/BR/2006/HUB-041621 | BR-DF | Brasília |
| 2 | 'TYPE18' | 3052 | MHOM/BR/2008/RN-03 | BR-RN | Natal |
| 2 | 'TYPE18' | 3109 | MHOM/BR/2009/HU-UFS04 | BR-SE | Aracaju |
| 2 | 'TYPE19' | 906 | MHOM/BR/1987/H-136 | BR-CE | Boa Viagem |
| 2 | 'TYPE19' | 3173 | MCAN/BR/2010/LILICA II | BR-MT | Cuiabá |
| 3 | 'TYPE20' | 2581 | MHOM/BR/2003/JHS | BR-MS | Campo Grande |
| 3 | 'TYPE20' | 2584 | MHOM/BR/2003/JBIC | BR-MS | Campo Grande |
| 3 | 'TYPE21' | 2572 | MHOM/BR/2003/ALX | BR-MS | Três Lagoas |
| 3 | 'TYPE21' | 2697 | MHOM/BR/2004/phufms-122 | BR-MS | Campo Grande |
| 3 | 'TYPE22' | 600 | MHOM/BR/1985/CCC-H28 | BR-CE | Aquiraz |
| 3 | 'TYPE22' | 2561 | MHOM/BR/2003/AAS | BR-MS | Campo Grande |
| 3 | 'TYPE22' | 2570 | MHOM/BR/2003/VC | BR-MS | Nioaque |
| 3 | 'TYPE22' | 2575 | MHOM/BR/2003/RMJ | BR-MS | Campo Grande |
| 3 | 'TYPE22' | 2576 | MHOM/BR/2003/DDG | BR-MS | Campo Grande |
| 3 | 'TYPE22' | 2591*^§^* | MHOM/BR/2003/CAS | BR-MS | Bodoquena |
| 3 | 'TYPE22' | 2594 | MHOM/BR/2003/FCM | BR-MS | Jardim |
| 3 | 'TYPE22' | 2648 | MHOM/BR/2003/GJR | BR-MS | Campo Grande |
| 3 | 'TYPE22' | 2651*^§^* | MHOM/BR/2003/MAM | BR-MS | Campo Grande |
| 3 | 'TYPE22' | 2664*^§^* | MCAN/BR/2002/LVV-135 | BR-MS | Campo Grande |
| 3 | 'TYPE22' | 2701 | MHOM/BR/2003/phufms-146 | BR-MS | Campo Grande |
| 3 | 'TYPE22' | 2702 | MHOM/BR/2003/phufms-155 | BR-MS | Três Lagoas |
| 3 | 'TYPE22' | 2703 | MHOM/BR/2003/phufms-217 | BR-MS | Campo Grande |
| 3 | 'TYPE22' | 2704 | MHOM/BR/2004/phufms-11 | BR-MS | Campo Grande |
| 3 | 'TYPE22' | 2749 | MHOM/BR/2005/phufms-89 | BR-MS | Campo Grande |
| 3 | 'TYPE22' | 2778 | MHOM/BR/2005/phufms-89 | BR-MS | Campo Grande |
| 3 | 'TYPE22' | 2935 | MHOM/BR/2007/ARL | BR-MS | Campo Grande |
| 3 | 'TYPE22' | 3035 | MCAN/BR/2007/CG-2 | BR-MS | Campo Grande |
| 1 | 1 | 2506*^§^* | MCAN/BR/2001/CP-SEMNOME | BR-ES | Pancas |
| 1 | 2 | 2504*^§^* | MHOM/BR/2001/HP-EMO | BR-ES | Pancas |
| 1 | 5 | 2543 | MCAN/BR/2002/RN-CEPA2 | BR-RN | Natal |
| 1 | 7 | 3081 | MHOM/BR/2006/HUB-130597-MOR4 | BR-DF | Brasília |
| 1 | 10 | 3118 | MHOM/BR/2009/BLVD | BR-AM | Manaus |
| 1 | 11 | 3063 | MCAN/BR/2008/CAD-03 | BR-ES | Água Doce do Norte |
| 1 | 13 | 3106 | MHOM/BR/2009/HU-UFS01 | BR-SE | Aracaju |
| 1 | 14 | 3194 | MCAN/BR/2010/LAILA | BR-RS | Uruguaiana |
| 1 | 15 | 3125 | MHOM/BR/2009/VCF | BR-MA | São Luís |
| 1 | 16 | 667*^§^* | MHOM/BR/1986/H-33 | BR-CE | Pentecoste |
| 1 | 19 | 2060 | MHOM/BR/1995/MARIELSON | BR-BA | Jequié |
| 1 | 21 | 3187 | MCAN/BR/2010/PV75 | BR-RN | Natal |
| 1 | 23 | 3174 | MCAN/BR/2010/LARA I | BR-RS | Santa Maria |
| 1 | 24 | 3131 | MHOM/BR/2009/DAIANE-MO | BR-MT | Cuiabá |
| 1 | 26 | 2433 | MCAN/BR/1999/LC147J99 | BR-BA | Jequié |
| 1 | 28 | 603 | MCAN/BR/1984/CCC-17.481 | BR-CE | Caucaia |
| 1 | 29 | 2901 | MCAN/BR/2006/NMT-DF1421MO | BR-DF | Brasília |
| 1 | 30 | 2902 | MCAN/BR/2005/NMT-DF159MO | BR-DF | Brasília |
| 1 | 31 | 2898 | MHOM/BR/2006/NMT-HUB402982MO | BR-DF | Brasília |
| 1 | 33 | 3177 | MCAN/BR/2010/PV64 | BR-RN | Natal |
| 1 | 35 | 2544 | MCAN/BR/2002/RN-CEPA3 | BR-RN | Natal |
| 1 | 36 | 246 | MDID/BR/1983/CPQGM22 | BR-BA | Jacobina |
| 2 | 40 | 2061 | MHOM/BR/1995/MERIVALDO | BR-BA | Jequié |
| 2 | 41 | 634 | MCAN/BR/1984/CPQGM85 | BR-BA | Conde |
| 2 | 43 | 3114 | MHOM/BR/2009/HU-UFS09 | BR-SE | Aracaju |
| 2 | 44 | 3138 | MCAN/BR/2009/BRONCRIS | BR-MT | Rondonópolis |
| 2 | 45 | 3136 | MCAN/BR/2009/PATETA | BR-MT | Rondonópolis |
| 2 | 46 | 3134 | MCAN/BR/2009/GRANDÃO I | BR-MT | Rondonópolis |
| 2 | 47 | 3189 | MCAN/BR/2010/BURRINHO I | BR-RS | Uruguaiana |
| 2 | 49 | 3212 | MCAN/BR/2010/DIMY I | BR-MT | Cuiabá |
| 2 | 50 | 3224 | MCAN/BR/2010/GUG | BR-MT | Cuiabá |
| 2 | 51 | 3219 | MCAN/BR/2010/TITÃ I | BR-MT | Cuiabá |
| 2 | 52 | 3211 | MCAN/BR/2010/CALISTO II | BR-MT | Cuiabá |
| 2 | 53 | 2896 | MCER/BR/1979/M6445 | BR-PA | Salvaterra |
| 2 | 54 | 1177 | MHOM/BR/1988/BA-152 | BR-BA | Jacobina |
| 2 | 55 | 2683 | MHOM/BR/2002/LDV | BR-MS | Ribas do Rio Pardo |
| 3 | 56 | 2666 | MCAN/BR/2002/LVV-137 | BR-MS | Três Lagoas |
| 3 | 57 | 3111 | MHOM/BR/2009/HU-UFS06 | BR-SE | Aracaju |
| 3 | 58 | 1848*^§^* | MCAN/BR/1991/1194BAJE | BR-BA | Jequié |
| 3 | 59 | 3051 | MHOM/BR/2008/RN-05 | BR-RN | Natal |
| 3 | 60 | 2686 | MHOM/BR/2002/BGC | BR-MS | Três Lagoas |
| 3 | 62 | 2698 | MHOM/BR/2004/phufms-139 | BR-MS | Anastácio |
| 3 | 64 | 2649 | MHOM/BR/2003/JT | BR-MS | Aquidauana |
| 3 | 65 | 2705 | MHOM/BR/2004/phufms-149 | BR-MS | Campo Grande |
| 3 | 67 | 2566 | MHOM/BR/2003/MMV | BR-MS | Cipolândia |

*^§^*, strains previously analized by Kuhls et al. [4].

A description of the International Code for labeling *Leishmania* strains is available in TRS 949 (WHO, 2010). International Code: M, Mammalian; HOM, human (*Homo*); CAN, dog (*Canis*); DID, opossum (*Didelphis*); CER, fox (*Cerdocyon*); BR, Brazil; PY, Paraguay

State codes (ISO 3166-2): BR-AM, Amazonas; BR-BA, Bahia; BR-CE, Ceará; BR-DF, Distrito Federal; BR-ES, Espírito Santo; BR-MA, Maranhão; BR-MG, Minas Gerais; BR-MT, Mato Grosso; BR-MS, Mato Grosso do Sul; BR-PA, Pará; BR-PE, Pernambuco; BR-PI, Piauí; BR-RJ, Rio de Janeiro; BR-RN, Rio Grande do Norte; BR-RS, Rio Grande do Sul; BR-SE, Sergipe; BR-SP, São Paulo; PY-ASU, Asunción
