# Supplementary material for: The Genetic Structure of Leishmania infantum Populations in Brazil and Its Possible Association with the Transmission Cycle of Visceral Leishmaniasis
Source: PLoS One. 2012 May 11;7(5):e36242. doi: 10.1371/journal.pone.0036242 (PMC3350531; doi:10.1371/journal.pone.0036242)
Supplement: Table S2 — Descriptive statistics of the Leishmania infantum MLMT profiles of populations from STRUCTURE analysis. (DOC) [file pone.0036242.s004.doc]

**Table S2. Descriptive statistics of the *Leishmania infantum* MLMT profiles of populations from STRUCTURE analysis**.

| **Locus** | **Pop ID** | **N** | **Repeat array** | **Size array** | **A** | ***H*_o_** | ***H*_e_** | ***F*_IS_** |
| --- | --- | --- | --- | --- | --- | --- | --- | --- |
| **Lm2TG (L)** | POP1 | 111 | 25/30 | 142/152 | 3 | 0 | 0.1955 | 1 |
|  | POP2 | 31 | 23/27 | 138/146 | 4 | 0.1935 | 0.5865 | 0.6736 |
|  | POP3 | 31 | 23/29 | 138/150 | 6 | 0.1290 | 0.2909 | 0.5604 |
| **TubCA (T)** | POP1 | 111 | 9/10 | 80/82 | 2 | 0 | 0.0528 | 1 |
|  | POP2 | 30 | 9/9 | 80/80 | 1 | 0 | 0 | 0 |
|  | POP3 | 31 | 9/9 | 80/80 | 1 | 0 | 0 | 0 |
| **Lm4TA (M)** | POP1 | 108 | 11/13 | 77/81 | 3 | 0.0093 | 0.0457 | 0.7981*^ns^* |
|  | POP2 | 31 | 11/14 | 77/83 | 4 | 0.2581 | 0.5706 | 0.5518 |
|  | POP3 | 28 | 12/13 | 79/81 | 2 | 0.1071 | 0.1032 | -0.0385*^ns^* |
| **Li41-56 (B)** | POP1 | 108 | 10/11 | 90/92 | 2 | 0 | 0.0184 | 1 |
|  | POP2 | 31 | 10/10 | 90/90 | 1 | 0 | 0 | 0 |
|  | POP3 | 31 | 10/10 | 90/90 | 1 | 0 | 0 | 0 |
| **Li46-67 (C)** | POP1 | 110 | 9/10 | 80/82 | 2 | 0.0182 | 0.0181 | -0.0046*^ns^* |
|  | POP2 | 31 | 9/9 | 80/80 | 1 | 0 | 0 | 0 |
|  | POP3 | 31 | 9/9 | 80/80 | 1 | 0 | 0 | 0 |
| **Li22-35 (E)** | POP1 | 106 | 12/16 | 92/100 | 4 | 0.0189 | 0.2054 | 0.9086 |
|  | POP2 | 28 | 13/13 | 94/94 | 1 | 0 | 0 | 0 |
|  | POP3 | 31 | 13/14 | 94/96 | 2 | 0 | 0.0635 | 1 |
| **Li23-41 (F)** | POP1 | 104 | 12/17 | 77/87 | 3 | 0 | 0.0749 | 1 |
|  | POP2 | 28 | 16/16 | 85/85 | 1 | 0 | 0 | 0 |
|  | POP3 | 30 | 16/16 | 85/85 | 1 | 0 | 0 | 0 |
| **Li45-24 (G)** | POP1 | 110 | 3/16 | 81/107 | 4 | 0.0091 | 0.0797 | 0.8863 |
|  | POP2 | 31 | 15/16 | 105/107 | 2 | 0 | 0.2285 | 1 |
|  | POP3 | 31 | 15/15 | 105/105 | 1 | 0 | 0 | 0 |
| **Li71-33 (P)** | POP1 | 110 | 10/11 | 103/105 | 2 | 0.0182 | 0.0533 | 0.6599*^ns^* |
|  | POP2 | 30 | 11/12 | 105/107 | 2 | 0.0333 | 0.0966 | 0.6588*^ns^* |
|  | POP3 | 31 | 11/12 | 105/107 | 2 | 0 | 0.0635 | 1 |
| **Li71-5/2 (Q)** | POP1 | 111 | 9/9 | 110/110 | 1 | 0 | 0 | 0 |
|  | POP2 | 31 | 9/9 | 110/110 | 1 | 0 | 0 | 0 |
|  | POP3 | 31 | 9/9 | 110/110 | 1 | 0 | 0 | 0 |
| **Li71-7 (R)** | POP1 | 109 | 12/14 | 98/102 | 3 | 0.0183 | 0.0183 | -0.0023*^ns^* |
|  | POP2 | 31 | 13/13 | 100/100 | 1 | 0 | 0 | 0 |
|  | POP3 | 31 | 13/13 | 100/100 | 1 | 0 | 0 | 0 |
| **CS20 (S)** | POP1 | 111 | 18/18 | 83/83 | 1 | 0 | 0 | 0 |
|  | POP2 | 31 | 18/18 | 83/83 | 1 | 0 | 0 | 0 |
|  | POP3 | 31 | 18/18 | 83/83 | 1 | 0 | 0 | 0 |
| **kLIST7031 (K)** | POP1 | 107 | 11/11 | 111/111 | 1 | 0 | 0 | 0 |
|  | POP2 | 29 | 11/11 | 111/111 | 1 | 0 | 0 | 0 |
|  | POP3 | 31 | 11/11 | 111/111 | 1 | 0 | 0 | 0 |
| **kLIST7039 (I)** | POP1 | 110 | 14/15 | 205/207 | 2 | 0 | 0.0533 | 1 |
|  | POP2 | 29 | 15/15 | 207/207 | 1 | 0 | 0 | 0 |
|  | POP3 | 29 | 15/17 | 207/211 | 2 | 0 | 0.0678 | 1 |
| **Overall** | POP1 | 109 | - | - | 2 | 0.0066 | 0.0582 | 0.8877 |
|  | POP2 | 30 | - | - | 2 | 0.0346 | 0.1059 | 0.6764 |
|  | POP3 | 31 | - | - | 2 | 0.0169 | 0.0421 | 0.6032 |

*n*, the number of samples per locus; A, the number of alleles per locus; *H*_o_, the observed heterozygosity; *H*_e_, the expected heterozygosity; *F*_IS_, the inbreeding coefficient; *^ns^*, non-significant values under confidence interval of 95%.
